# Termination of figure-of-eight reentry via resonant feedback pacing

**DOI:** 10.3389/fnetp.2025.1692372

**Published:** 2025-11-19

**Authors:** Navneet Roshan, Rupamanjari Majumder

**Affiliations:** Nantes Université, CNRS, INSERM, l’institut du thorax, Nantes, France

**Keywords:** arrhythmia, feedback pacing, defibrillation, synchronization, subthreshold stimulation, spiral waves

## Abstract

Sudden cardiac death (SCD) is often precipitated by reentrant arrhythmias such as ventricular tachycardia (VT) and ventricular fibrillation (VF), whose underlying dynamics are frequently sustained by spiral waves of electrical activity. Disrupting these waves can restore normal rhythm, but conventional low-energy pacing strategies are often ineffective in VF, where high-frequency, multi-wave interactions dominate. Resonant feedback-controlled antitachycardia pacing (rF-ATP), which times global electrical stimuli based on real-time feedback from the tissue, has been shown to robustly terminate single spirals under diverse conditions. However, its impact on interacting spiral waves—arguably a more realistic substrate for life-threatening arrhythmias—remains unexplored. Here, we use numerical simulations to investigate the effect of rF-ATP on figure-of-eight reentry, a clinically relevant configuration consisting of two counter-rotating spirals. We show that rF-ATP consistently terminates this pattern, regardless of feedback point location, through two distinct dynamical pathways: mutual collision of phase singularities or annihilation at inexcitable boundaries. We further demonstrate the method’s efficacy across variations in feedback point and spiral arrangement, indicating robustness to geometrical and positional heterogeneity. These results highlight rF-ATP as a promising low-energy intervention for complex reentrant structures and provide mechanistic insight into feedback-driven control of multi-core spiral wave dynamics in cardiac tissue.

## 1 Introduction

Sudden cardiac death (SCD) remains one of the leading causes of mortality in the industrialized world (Mehra, 2007). A key precursor to SCD is the onset of life-threatening arrhythmias such as ventricular tachycardia (VT) and ventricular fibrillation (VF). These rhythm disturbances can emerge from a wide array of pathophysiological mechanisms across biological scales—from genetic mutations at the molecular level to structural abnormalities at the organ level (A. L. George, 2003; Veldkamp and Bezzina, 2020; Binas et al., 2020; Abriel, 2010; Shy et al., 2013; Saffitz, 2009; Peters and Wit, 2000). Despite this diversity of origin, many arrhythmias converge on a common electrophysiological manifestation: reentrant electrical activity, typically in the form of spiral excitation waves (Davidenko et al., 1990; Pandit and Jalife, 2013; Moe, 2005). These self-sustaining waves disrupt the heart’s intrinsic pacemaking system and dominate its electrical behavior, leading to compromised function and, in severe cases, SCD.

The dynamics of spiral waves have therefore long been a central focus of cardiac electrophysiology research, with the specific aim of achieving their targeted suppression to ensure electrical resynchronization and to restore normal rhythm. Consequently, understanding and controlling spiral waves represents a potentially powerful therapeutic strategy, especially for acute intervention in VT and VF. To this end, multiple approaches have been proposed (Santini et al., 2010; Nichol et al., 2017), including, the use of pharmacological agents (Wiedmann and Schmidt, 2024), optogenetic modulation (Majumder et al., 2018), low-energy electrical pacing (Sivagangabalan et al., 2013) etc. From a clinical perspective, low-amplitude electrical pacing remains the most viable, minimally invasive strategy for acute arrhythmia control. While such pacing has shown considerable success in managing VT, its effectiveness is limited in VF, primarily because multiple excitation frequencies can coexist during VF some of which may exceed and thereby suppress the externally applied pacing frequency, rendering it ineffective (Estes III et al., 1994; Strik et al., 2019).

To address this limitation, Biktashev and Holden (1994) proposed resonant feedback-controlled antitachycardia pacing (rF-ATP) a method where the timing of global electrical stimulation is determined by real-time electrical feedback from the tissue itself. This technique has been shown to eliminate single spiral waves across a broad range of conditions, regardless of the spiral tip trajectory or tissue parameters. However, rF-ATP has not yet been explored in the context of interacting spiral waves, which may represent a prominent class of realistic and clinically relevant arrhythmias.

Spiral waves in the heart can manifest as single or multiple rotating patterns, either synchronized or desynchronized, and with uniform or variable rotation frequencies (Vidmar et al., 2017; Mulimani et al., 2022; Weiss et al., 2000). Monomorphic ventricular tachycardia (VT) is typically associated with a single, periodically rotating spiral, whereas polymorphic VT may involve a drifting spiral or a spiral with variable frequency or multiple synchronized spirals (Davidenko, 1993). The most complex state—ventricular fibrillation (VF)—is characterized by multiple, asynchronous spirals interacting across the myocardium (Panfilov and Pertsov, 2001), posing major challenges for both clinical management and mechanistic understanding of the underlying nonlinear dynamics.

Figure-of-eight reentry (Gaztañaga et al., 2012; El-Sherif et al., 1981) is a canonical reentrant activation pattern consisting of two counter-rotating (clock and anti-clockwise) spirals anchored to adjacent anatomical or functional obstacles (Nishimura et al., 2021; Banville et al., 1999). This configuration creates a stable loop of excitation that can override normal pacemaking and sustain abnormal rhythms over extended periods. It is frequently observed in clinical electrophysiology (Wu et al., 1994; Lin et al., 1999; Roshan and Pandit, 2023), particularly after myocardial infarction (Nishimura et al., 2021), where scar tissue and border zones provide the substrate for wave anchoring. Moreover, the functional conduction block in intact cardiac tissue can also produce two counter-rotating spirals, resembling the figure-of-eight reentry (Wu et al., 1994; Roth and Saypol, 1991; Banville et al., 1999; Martinez-Navarro et al., 2021). Its stability and reproducibility make it an important model for studying the initiation, maintenance, and termination of reentry (Okada et al., 2021), as well as for developing and optimizing interventions such as catheter ablation and pacing therapies. From a physics perspective, figure-of-eight reentry represents a controlled system for probing interactions between spatially organized wave sources and the global cardiac activation network, and for exploring how targeted perturbations can restore synchrony.

In this study, we investigate the dynamics of figure-of-eight reentry and its susceptibility to rF-ATP. We show how this phase-sensitive feedback intervention can terminate reentry through two main pathways: (i) direct annihilation via in-phase spiral collision, and (ii) repulsion-driven drift toward domain boundaries, followed by phase singularity annihilation. Our findings provide mechanistic insights into how feedback control can disrupt multi-core reentrant structures and point toward new therapeutic strategies for terminating complex ventricular arrhythmias.

## 2 Materials and methods

### 2.1 Cardiac tissue model

All simulations were conducted using the three-variable Fenton-Karma (FK) model, a phenomenological model of cardiac electrophysiology designed to reproduce essential features of ventricular action potentials with reduced computational complexity. The FK model consists of a scaled transmembrane voltage variable u , with two other variable v and w . The governing Equations 1-3:

$$\partial_{t}u=\nabla \cdot \left(D\nabla u\right)-J_{\text{fi}}\left(u;v\right)-J_{\text{so}}\left(u\right)-J_{\text{si}}\left(u;w\right),$$ (1)

$$\partial_{t}v=\Theta \left(u_{c}-u\right)\left(1-v\right)/\tau_{v}^{-}\left(u\right)-\Theta \left(u-u_{c}\right)v/\tau_{v}^{+},$$ (2)

$$\partial_{t}w=\Theta \left(u_{c}-u\right)\left(1-w\right)/\tau_{w}^{-}-\Theta \left(u-u_{c}\right)w/\tau_{w}^{+},$$ (3)

where the three currents are given by Equations 4-6:

$$J_{\text{fi}}\left(u;v\right)=-\frac{v}{\tau_{d}}\Theta \left(u-u_{c}\right)\left(1-u\right)\left(u-u_{c}\right),$$ (4)

$$J_{\text{so}}\left(u\right)=\frac{u}{\tau_{o}}\Theta \left(u_{c}-u\right)+\frac{1}{\tau_{r}}\Theta \left(u-u_{c}\right),$$ (5)

$$J_{\text{si}}\left(u;w\right)=-\frac{w}{2\tau_{\text{si}}}\left(1+\tanh\left[k\left(u-u_{c}^{\text{si}}\right)\right]\right),$$ (6)

where $J_{\text{fi}}$ , $J_{\text{so}}$ and $J_{\text{si}}$ represents the fast inward, slow outward, and slow inward components. Parameter values follow the modified forms of the (Beeler and Reuter, 1977) parameter set of the original Fenton-Karma model [see Ref. Fenton and Karma (1998)], which supports the formation and sustainability of spiral waves. To recover the transmembrane voltage V in mV from variable u we use the mapping V=100⋅u−85 . The parameter values are k=10 , $u_{c}=0.13$ , $\tau_{v}^{+}=3.33$ , $\tau_{w}^{-}=11$ , $\tau_{w}^{+}=667$ , $g_{{\text{fi}}_{\text{max}}}=2.4$ , $\tau_{d}=1/g_{{\text{fi}}_{\text{max}}}$ , $\tau_{0}=8.3$ , $\tau_{r}=50$ , $\tau_{\text{si}}=45$ , $\tau_{v}^{-}\left(u\right)=\Theta \left(u-u_{v}\right)\tau_{v1}^{-}+\Theta \left(u_{v}-u\right)\tau_{v2}^{-}$ with $u_{v}=0.055$ , $\tau_{v1}^{-}=1000$ , $\tau_{v2}^{-}=19.2$ , with Θ as the Heaviside’s function.

The diffusion coefficient D=1 cm^2^ s^−1^ was chosen to yield physiologically realistic conduction velocities (67 cm/s). The computational domain was modeled as a 2D grid ( 768×768 nodes) with isotropic diffusion and Neumann (no-flux) boundary conditions.

### 2.2 Numerical implementation

Numerical integration was performed using a forward Euler scheme with a time step Δt=0.05 ms and spatial resolution Δx=0.02 cm. The Laplacian operator was discretized using a five-point finite difference stencil. Simulations were run for up to 5000 m of model time to capture both transient and steady-state spiral dynamics.

Voltage traces, tip trajectories, and phase singularities were analyzed using custom MATLAB scripts, and (Ahrens et al., 2005) was used for the postprocessing and visualizations. Reentry termination was defined as the disappearance of all phase singularities within the simulation domain.

### 2.3 Spiral wave initiation

**FIGURE 1:**
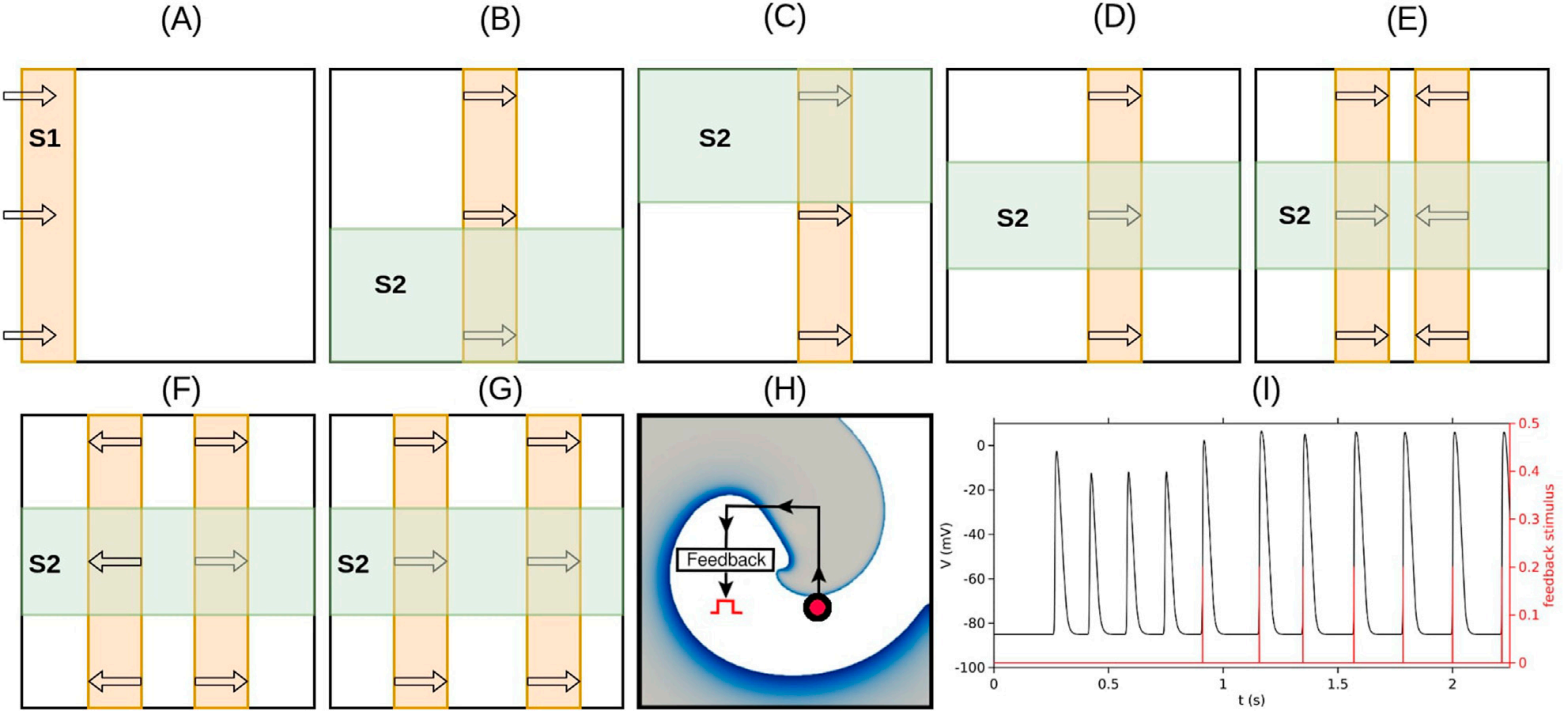
*Schematic illustration of the stimulation protocols used to generate various spiral wave patterns* and provide feedback pacing. **(A)** Initiation of the S1 wave (orange) via a line stimulus applied along the left edge of the simulation domain. **(B)** S2 stimulus (green) applied to the lower half of the domain when the waveback of the S1 wave reaches the midpoint, resulting in a clockwise-rotating spiral. **(C)** S2 stimulus applied to the upper half of the domain at the same waveback position produces a counterclockwise-rotating spiral. **(D)** S2 stimulus applied over a rectangular region away from the domain boundaries produces a stable figure-of-eight pattern. **(E)** Protocol for generating four spiral waves (two face-to-face figure-of-eight patterns): two S1 waves are initiated simultaneously by applying line stimuli along both the left and right edges of the domain; the S2 stimulus is applied as in **(D)**. **(F)** Protocol for generating four spiral waves (two back-to-back figure-of-eight patterns): a single S1 wave is initiated by applying a vertical line stimulus along the center of the domain; the S2 stimulus is applied as in **(D)**. **(G)** Protocol for generating four spiral waves (two figure-of-eight patterns of the same chirality): two S1 waves are initiated sequentially by applying line stimuli along the left edge of the domain; the S2 stimulus is applied as in **(D)**. **(H)** Schematic of the resonant feedback pacing (rF-ATP) protocol: the feedback point is marked with a red circle; each time the voltage at this point overshoots a threshold, a global stimulus is applied. **(I)** Voltage trace (black) and feedback stimulus timings (red) for a representative case of rF-ATP application.

Spiral waves were initiated using a classical S1–S2 cross-field protocol. The S1 stimulus was applied as a planar wavefront from one edge of the domain. After the wavefront traversed half the domain, an S2 stimulus was applied orthogonally to a defined rectangular region. This led to wavefront breakup and the formation of spiral patterns. Modifications in the S2 geometry enabled the generation of single spirals, figure-of-eight configurations, or more complex four-spiral states, as illustrated schematically in Figures 1A–G.

### 2.4 Resonant feedback anti-tachycardia pacing (rF-ATP)

Resonant feedback antitachycardia pacing (rF-ATP) was implemented to study reentrant wave control. The pacing protocol relied on real-time electrical feedback from the tissue, as depicted in the schematic of Figure 1H. A measuring electrode was positioned at a selected location within the simulation domain (indicated by the filled red circle in Figure 1H), hereafter referred to as the feedback point. Each time the voltage at the feedback point crossed a preset threshold ( ∼0.5 in non-dimensional units), a subthreshold global stimulus $I_{\text{stim}}<=0.2$ pA pF^−1^ was applied to the entire domain, for the duration of 0.5 m. The threshold for stimulation occurred at 0.3 pA pF^−1^ for the same duration of the applied stimulus. The voltage time series recorded at the feedback point (black) is shown in Figure 1I, together with the applied feedback signal (red). The applied stimulus was designed to modulate the excitability of the domain and entrain ongoing activity through phase interactions. To evaluate spatial sensitivity, various locations were tested for the feedback site, and both clockwise and counterclockwise spirals were simulated to assess the effect of chirality.

### 2.5 Calculating spiral tip trajectories

To identify the tip (phase singularity) of the spiral wave, we follow the approach described in Ref. Iyer and Gray (2001). During excitation, the state variable u(x,y,t) at any spatial point (x,y) rises from its resting value, reaches a peak, and subsequently returns to rest. To uniquely characterize the local state, we define the *phase* variable as per Equation 7:

$$\theta \left(x,t\right)=\arctan2\left[\;u\left(x,t\right)-u^{*},\;u\left(x,t-\tau_{0}\right)-u^{*}\;\right],$$ (7)

where $\tau_{0}=5$ ms time units (t.u.) corresponds to 100 time steps with Δt=0.05 ms, and $u^{*}$ denotes the spatial average of u(x,y,t) . The spiral tip is located at points where all iso-contours of θ converge and where θ varies smoothly from 0 to 2π around the point. The position of the phase singularity is determined using Equation 8:

∮∇θ⋅dr=±2π. (8)

## 3 Results

### 3.1 Dynamics of a single spiral wave in the presence of resonant feedback pacing

To develop a mechanistic understanding of how resonant feedback antitachycardia pacing (rF-ATP) influences a pair of counter-rotating spiral waves—particularly in the case of figure-of-eight reentry-it is first useful to examine the response of an isolated spiral wave under identical conditions.

**FIGURE 2:**
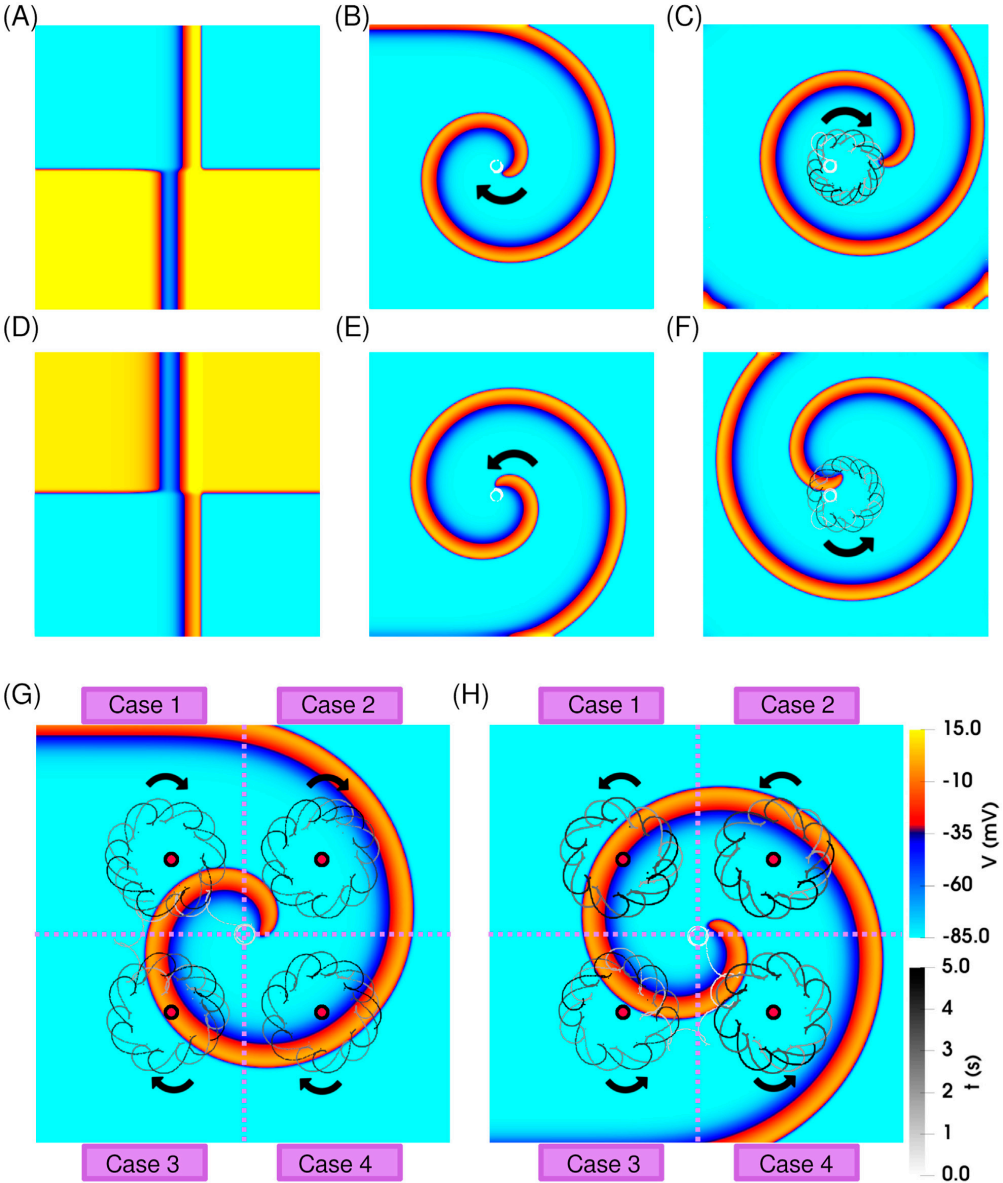
*Dynamics of a single spiral wave in the absence and presence of resonant feedback antitachycardia pacing (rF-ATP).* **(A)** Spatial distribution of transmembrane potential at the moment of S2 stimulation, producing a clockwise-rotating spiral wave. **(B)** Stable spiral wave observed after 5 s of simulation; the tip trajectory is overlaid in white, showing a circular path. **(C)** Application of rF-ATP using feedback from the geometric center of the domain results in spiral precession around the feedback site, forming an epicycloidal tip trajectory. **(D–F)** Corresponding results for a counterclockwise-rotating spiral: **(D)** S2 stimulation pattern, **(E)** stable spiral without feedback, and **(F)** spiral dynamics under rF-ATP, showing reversed epicycloidal precession. Dynamics of a single **(G)** clockwise-rotating spiral, and **(H)** counterclockwise-rotating spiral wave subjected to rF-ATP. Electrical signals were recorded from four distinct feedback sites (marked by red circles), each located in a different quadrant of the simulation domain. Regardless of feedback location, rF-ATP consistently induced spiral precession around the corresponding site, with the same sense of rotation relative to spiral chirality.

To initiate an isolated clockwise-rotating spiral wave, we applied a standard S1–S2 cross-field protocol, as explained in Section 2.3 and illustrated in Figure 2A. Over time, this excitation pattern evolved into a solitary, unperturbed spiral, rotating clockwise with a stable, circular tip trajectory (shown in white) on Figure 2B.

After an initial transient of approximately five stable rotations, the rF-ATP protocol was initiated. When feedback was extracted from the geometric center of the domain, rF-ATP induced a stable precessional motion of the spiral around the feedback point, following an epicycloidal tip trajectory, as shown in Figure 2C. This behaviour is consistent with the classical response observed under rF-ATP. A spiral with opposite chirality (counterclockwise rotation) also exhibited similar dynamics with an expected reversal in the sense of rotation; corresponding results are presented in Figures 2D–F. Moreover, to ensure that the observed spiral wave dynamics were not biased by the specific location of the feedback site, we repeated the simulations using four different feedback positions across the simulation domain [see Figures 2G,H]. While the transient responses varied depending on the proximity of the feedback site and the amplitude of the applied stimulation, the overall behavior remained consistent: in all cases, rF-ATP successfully induced drift of the spiral tip toward the feedback location, followed by stable precession. This outcome aligns with the expected dynamics of classical resonant feedback based pacing Hussaini et al. (2023) in the chosen parameter regime for both spiral orientations. Importantly, the pacing response was symmetric with respect to chirality, indicating that no inherent bias arises from the direction of rotation in the absence of interactions.

### 3.2 Dynamics of a figure-of-eight pattern in the presence of resonant feedback pacing

**FIGURE 3:**
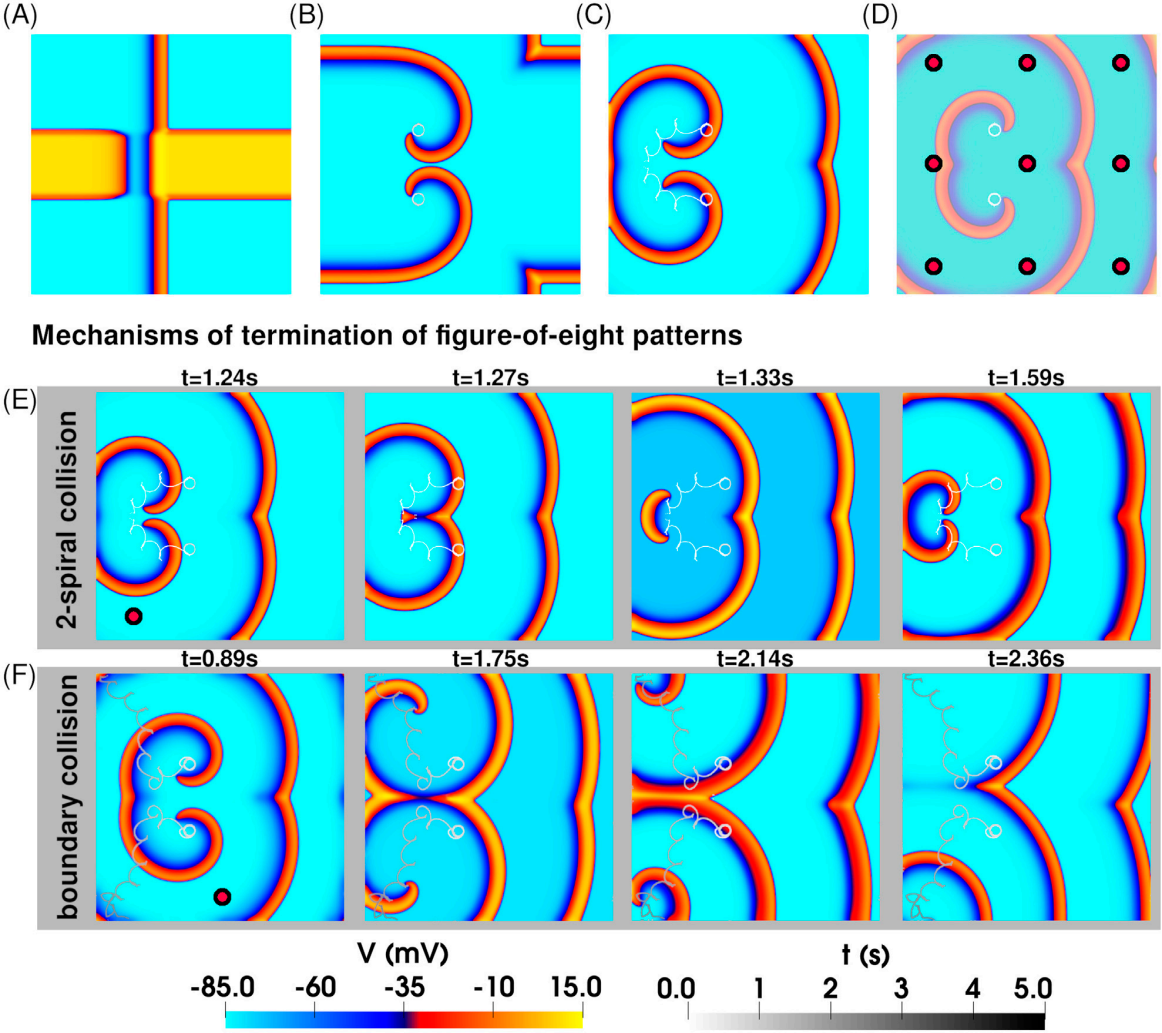
*Application of rF-ATP to a pair of interacting spiral waves in a figure-of-eight pattern leads to termination.* **(A)** Spatial distribution of transmembrane potential at the moment of S2 stimulation, producing a figure-of-eight pattern with two spirals of opposite chirality. **(B)** Stable figure-of-eight configuration, with each spiral tip tracing a circular trajectory. **(C)** Application of rF-ATP modifies spiral–spiral interactions, leading to termination—here via mutual collision and subsequent annihilation of the phase singularities. **(D)** Nine feedback points (marked by red circles) used to test the rF-ATP protocol on the same initial figure-of-eight pattern. In all cases, termination occurred via one of two mechanisms illustrated in **(E)** and **(F)**. **(E)** Termination via mutual collision of the two phase singularities: Representative case in which rF-ATP induced mutual attraction between the spirals. **(F)** Termination via collision of each phase singularity with the inexcitable boundary: Representative case in which rF-ATP caused the spirals to repel each other.

Next, we studied the dynamics of two interacting spiral waves. We generated a simple figure-of-eight pattern as per Figure 1D. This excitation pattern (Figure 3A) evolved in space and time to produce two coupled spirals forming a stable figure-of-eight configuration, where each spiral independently traced a circular tip trajectory, unperturbed by the presence of its partner [Figure 3B].

We then applied rF-ATP to this configuration, using electrical feedback from the geometric center of the simulation domain. Both spirals responded immediately to the applied stimulus, initiating a clear drift. The distance between the two spiral tips decreased monotonically until the phase singularities got annihilated via mutual collision, as illustrated in Figure 3C. To test for positional bias of the feedback site, we repeated the simulations using electrical feedback from nine different locations across the domain (indicated by red circles on Figure 3D). In all cases, we observed successful termination of the figure-of-eight pattern, albeit through distinct dynamical routes, demonstrating that rF-ATP is capable of terminating complex, interacting spiral-wave patterns.

Our studies demonstrate that the application of rF-ATP to a pair of stable, interacting spiral waves can result in one of two distinct outcomes: (a) annihilation of the two phase singularities located at the spiral tips through in-phase mutual collision, or (b) mutual repulsion between the spirals, ultimately causing one or both to drift toward and collide with a domain boundary, leading to termination. The second mechanism was observed in three out of nine simulations conducted. Since termination via boundary interaction is influenced by the precise nature of spiral-boundary dynamics—which can sometimes delay or even prevent annihilation due to repulsive effects—we propose that spiral termination through mutual collision offers a more robust and reliable mechanism for reestablishing electrical synchrony in the tissue with rF-ATP.

### 3.3 Dynamics of two interacting figure-of-eight patterns in the presence of resonant feedback pacing

**FIGURE 4:**
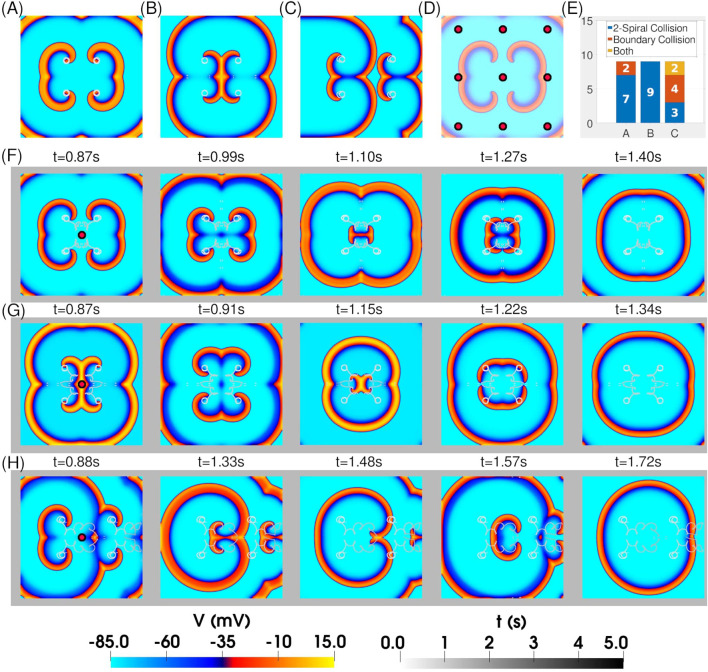
*Application of rF-ATP to two weakly interacting figure-of-eight patterns leads to termination.* Spatial distribution of transmembrane potential after stabilization of two figure-of-eight patterns that **(A)** face each other, **(B)** are back-to-back, and **(C)** are positioned side-by-side. In each configuration, the individual spirals exhibit stable circular tip trajectories in steady state. **(D)** Locations of nine feedback points used in separate simulations for each 4-spiral configuration in **(A)**–**(C)** to test for positional bias of the protocol. **(E)** Statistics of figure-of-eight termination via the two mechanisms described in Figure 3. **(F–H)** Representative spatiotemporal evolutions of the patterns in **(A**–**C)** when feedback is extracted from the geometric center of the domain. In all cases, rF-ATP leads to figure-of-eight termination via annihilation of mutually colliding phase singularities.

We next examined the response of two interacting figure-of-eight reentrant patterns (quatrefoil reentry) to resonant feedback antitachycardia pacing (rF-ATP). For these simulations, the S2 stimuli were chosen to generate spirals with well-separated cores, ensuring that each spiral could maintain its original circular tip trajectory in the absence of pacing. Three distinct figure-of-eight configurations were tested: (*i*) two mirror-inverted figure-of-eight patterns exhibiting head-on interactions (Figure 4A)– hereafter referred to as *case i*, (*ii*) two mirror-inverted figure-of-eight patterns exhibiting back-to-back interactions (Figure 4B)– hereafter referred to as *case ii*, and (*iii*) two adjacent figure-of-eights with the same chirality (Figure 4C)– hereafter referred to as *case iii*.

We then applied rF-ATP using feedback derived from nine distinct points across the simulation domain [Figure 4D]. In all configurations, the intervention successfully terminated the reentrant activity, although the termination mechanism depended on the feedback site. Figure 4E summarizes the distribution of termination mechanisms for each configuration. In quatrefoil reentry *case i*, termination occurred predominantly (7/9 cases) via in-phase collision of a pair of phase singularities—typically originating from the same figure-of-eight—resulting in mutual annihilation. In the remaining 2/9 cases, the spirals repelled each other, with their phase singularities drifting toward the inexcitable domain boundary, where they were subsequently eliminated. Quatrefoil reentry *case ii* predominantly terminated through 2-spiral collision, with no boundary-mediated elimination observed. In contrast, quatrefoil reentry *case iii* exhibited both termination mechanisms with approximately equal frequency.Representative spatiotemporal maps of transmembrane voltage for each configuration under rF-ATP, with feedback taken from the geometric center of the four-spiral system, are shown in Figures 4F–H.

## 4 Discussion

Figure-of-eight reentry is a fundamental pattern in excitable media characterized by two dynamically coupled spiral waves. The initiation of a pair of spirals with opposite chirality is associated with conservation of topological charges governing their creation and annihilation (Pertsov et al., 2000; Davidsen et al., 2004). Recent studies have extended this framework to complex geometries, revealing how such dynamics is expected to persist and evolve in the real heart (Marcotte and Grigoriev, 2017; Arno et al., 2024; Vandersickel et al., 2024; Arno et al., 2025; Tyree et al., 2024). Figure-of-eight reentry is particularly relevant in the context of cardiac arrhythmias like VT and VF (Banville et al., 1999; Lin et al., 1999; Iravanian et al., 2003; Janks and Roth, 2006; Martinez-Navarro et al., 2021). VF, for example, can be interpreted as the continuous generation, interaction, and annihilation of such pair of spirals (or phase defects) (Gil et al., 1990; Marcotte and Grigoriev, 2017; Tyree et al., 2024; Coullet et al., 1989; Duytschaever et al., 2024). Therefore, understanding the control and dynamics of figure-of-eight reentries is essential for advancing therapeutic approaches to life-threatening cardiac arrhythmias.

Previous studies, mainly using light-sensitive chemical reaction systems, have demonstrated that periodic modulation of excitability–implemented via time-dependent forcing or feedback signals–can induce spiral wave drift, suppress meander, anchor spiral cores, or destabilize reentrant activity to enable targeted wave termination (Steinbock et al., 1993; Grill et al., 1996; Kheowan et al., 2002; Kheowan and Müller, 2005; Braune and Engel, 2000; Zykov et al., 2003). In cardiac tissue, such feedback can be delivered electrically or optogenetically (Majumder et al., 2018; Xia et al., 2022), offering a less painful, minimally-invasive and spatiotemporally precise means of arrhythmia control. The mechanisms underlying these approaches involve both phase and frequency synchronization between the intrinsic rotation of the spiral wave and the externally applied periodic modulation. This interaction could restore the chaotic patterns to more ordered states, offering a powerful framework for dynamic control of excitable media.

In this study we demonstrate that rF-ATP: a low-amplitude, time-controlled electrical pacing protocol (Biktashev and Holden, 1994; Morgan et al., 2009), is capable of terminating both isolated and coupled figure-of-eight spiral wave patterns in cardiac tissue. We identified two primary mechanisms of spiral wave termination: (*i*) mutual annihilation via in-phase collision, and (*ii*) mutual repulsion, followed by annihilation at *inexcitable* domain boundaries. It is important to note that, in the present study, cardiac tissue boundaries were modeled using standard no-flux boundary conditions. No-flux boundaries may, sometimes, interact with spirals in a repulsive manner, causing them to reflect back (Olmos and Shizgal, 2008). This sort of interaction occurs when the spiral tip approaches the boundary tangentially or slowly, such that the curvature of the wavefront increases; the wavefront can then stall, deform or reflect off the boundary. Boundary effects on spiral wave dynamics (leading to their reflection) are more common in finite-size domains, where wavelength of the spiral is comparable to the domain size. In such domains, the no-flux boundary affects the field ahead of the wavefront before collision, sometimes leading to reflection. Our studies showed that in general, while both mechanisms (i and ii) can restore synchrony in the tissue, termination via mutual collision is more deterministic and robust, as boundary-mediated termination is influenced by spatial constraints and may be delayed or incomplete due to edge effects, or stemming from the fact that they do not explicitly enforce topological charge conservation, necessary for the annihilation of phase singularities. It is noteworthy that smaller domains are more susceptible to spiral wave reflection; however, they also tend to favor termination via mechanism *(i)*, as spatial constraints limit the coexistence of multiple spirals. In contrast, with increasing domain size, mechanism *(ii)* is expected to become more prominent, since phase singularities must traverse longer distances to interact with inexcitable boundaries. A systematic investigation into the influence of domain size on the relative prevalence and dynamics of these termination mechanisms would provide valuable insights but falls beyond the scope of the present work. In a recent study, DeTal et al. (2022) addressed this limitation by implementing charge-conserving ‘reflective’ boundaries—hypothetical boundaries within the simulation domain about which electrical activity is symmetric. This concept can be related to our individual spirals in the first two cases of quatrefoil reentry, if one assumes that the reflective boundaries’ coincide with the vertical and horizontal lines dividing the domain into four equal quadrants. Going by that notion, and based on our findings, one can conclude that rF-ATP can indeed terminate spirals in the presence of reflective boundaries which preserve topological charge conservation.

Another noteworthy aspect is that the results presented here were obtained with figure-of-eight patterns positioned sufficiently far apart to prevent significant mutual interactions in the absence of rF-ATP, thereby excluding additional effects such as spontaneous repulsion, precession, or self-termination. When the width of the S2 stimulus was adjusted to minimize the gap between the two spirals in a figure-of-eight pattern [Figures 1E–G], a variety of outcomes was observed. In most cases, without rF-ATP, mutual interactions led to spontaneous termination of one spiral pair, leaving a single figure-of-eight pattern in the domain. Application of rF-ATP to the remaining pair then achieved rapid and robust termination, essentially as observed for isolated figure-of-eight configurations. These results were confirmed for various inter-spiral distances and two additional subthreshold feedback amplitudes ( $I_{\text{stim}}=0.15$ pA pF^−1^ and $I_{\text{stim}}=0.1$ pA pF^−1^). A mechanistically similar approach was followed by Xia et al. (2022), who reported a feedback-driven termination strategy for spiral waves via mutual collision and annihilation of phase singularities in optogenetically modified cardiac tissue models. In their study, the applied feedback was not resonant, but instead involved a spatially non-uniform light pattern that preferentially depolarized the wavefront while simultaneously hyperpolarizing the waveback, thereby inducing a linear drift of the spiral core. This mechanism contrasts fundamentally with our approach, which employs spatially uniform, low-amplitude electrical stimulation applied in a resonant, phase-locked manner to signals extracted from a selected point within the simulation domain. Our findings support the notion that rF-ATP can act as a globally effective, non-specific control mechanism capable of disrupting highly complex and synchronized excitation patterns.

Taken together, our results reveal how rF-ATP interacts with complex spiral wave architectures, modulating isolated spirals and resolving multi-spiral interactions. This highlights its potential as a low-energy intervention for polymorphic VT and early-stage VF. The method’s robustness to feedback site and scalability to complex reentry patterns support its translational promise. Future studies should explore realistic tissue geometries, electromechanical coupling, heterogeneities, and stochastic variability, as achieving uniform stimulation across the myocardium is clinically challenging. In a true cardiac context, such modulation would be complex to implement due to virtual electrode effects, anatomical boundaries, scarring, and intrinsic heterogeneity of electrical conduction—all of which could influence the efficacy and implementation of rF-ATP. Integration with advanced sensing or hybrid (*in silico/in vitro*) platforms, and machine learning-driven adaptation of feedback parameters (Mulimani et al., 2020), could enable personalized, adaptive pacing therapies exploiting nonlinear cardiac dynamics for real-time arrhythmia control.

## Data Availability

The original contributions presented in the study are included in the article/supplementary material, further inquiries can be directed to the corresponding authors.
